# Nutritional, biochemical, and clinical applications of carob: A review

**DOI:** 10.1002/fsn3.3367

**Published:** 2023-06-09

**Authors:** Ali Ikram, Waseem Khalid, Khair‐ul‐ Wajeeha Zafar, Anwar Ali, Muhammad Faizan Afzal, Afifa Aziz, Izza Faiz ul Rasool, Ammar Al‐Farga, Faisal Aqlan, Hyrije Koraqi

**Affiliations:** ^1^ University Institute of Food Science and Technology, The University of Lahore Lahore Pakistan; ^2^ Department of Food Science, Faculty of Life Sciences Government College University Faisalabad Pakistan; ^3^ Department of Chemistry Government College University Faisalabad Pakistan; ^4^ Department of Epidemiology and Health Statistics, Xiangya School of Public Health Central South University Changsha China; ^5^ Department of Biochemistry, College of Sciences University of Jeddah Jeddah Saudi Arabia; ^6^ Department of Chemistry, College of Sciences Ibb University Ibb Governorate Yemen; ^7^ Faculty of Food Science and Biotechnology UBT‐Higher Education Institution Pristina Kosovo

**Keywords:** carob, economic, food application, limitations, medicinal, nutrition, polyphenol

## Abstract

Carob is botanically called as *Ceratonia siliqua* and belongs to the Legumes family. The fruit is derived from hermaphrodite trees and hard in shape. The carob contains high sugar contents in pulp, fat in seed and minerals like potassium, calcium, and phosphorus are present in pods. Polyphenols and antioxidants are abundant in leaves and pods. It can be used for enhancing human health due to its high nutritional profile. Carob gum is used in the pharmaceutical industry in the form of pomades, anti‐celiac ingredients, pills, and dental paste. The clinical carob can aid as an anti‐cancer, anti‐reflux, anti‐diabetic, anti‐diarrheal, anti‐hyperlipidemia, anti‐bacterial, anti‐microbial, and anti‐fungal. Nowadays, carob seeds are being used as an alternative to cocoa powder in food items whereas the leaves, pods, and seeds of carob are also historically used as food for animal feed. However, these parts of carob are available in markets with reasonable prices. Carob production, though with a rising contribution, contributes to the local economy. In this sense, we can incorporate knowledge on the chemical properties and the biological effect of carob fruits on human health. In this study, the supportive and health‐promoting impacts of carob are discussed along with the clinical testing obtained from natural constituents of carob. In addition, further studies can be performed to extract and separate polyphenols and antioxidant potential for the development of functional that play a valuable role in pharmaceutical and food sectors.

## Graphical abstract

In this study, along with the clinical testing obtained from natural constituents of carob. In addition, further development of appropriate approaches for the study of extraction and separation of polyphenols should be performed to determine their antioxidant potential for pharmaceutical and food sectors.

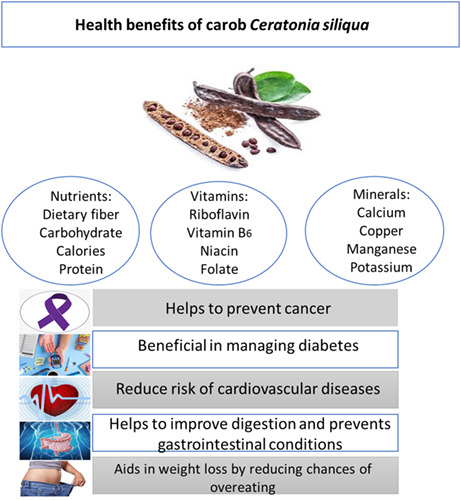

## 1 INTRODUCTION

*Ceratonia siliqua* L. is the botanical name of carob. It belongs to the plant family and derives from the Greek word “Kera.” These fruits have keratomorphic shapes which are the Latin word siliqua that refers to the pods' hardness and shape (Papaefstathiou et al., 2018). The carob tree (*C. siliqua* L.) is a native tree in the region of Mediterranean, and there occupies great importance on account of economic and environmental conditions (Ali, Kousar, et al., 2022; Benković et al., 2016). Carob fruit has a tough‐to‐crack pod that is elongated, flat, arced or straight, and thick sutures. There are edges that are either rounded or somewhat pointed. Its size ranges from 10 to 30 cm. Its composition is on two crucial parts including pulp (90%) and seeds (10%) (Durazzo et al., 2014). Cosmetics, food, and pharmaceutical industries use it widely as raw material (Iqra et al., 2023; Rasool et al., 2023). The latest research by Food and Agriculture Organization exposes that carob fruit is worldly produced at the rate of 158.609 tons per year, derived from 66,874 hectares, where the contribution from Asia, Europe, and Africa is 11.3%, 75.55%, and 13%, respectively (Pernet & Ribi Forclaz, 2019).

Carob is a representative fruit of the areas with the climate of the Mediterranean. The carob tree belongs to the legumes family, and evergreen tree with sensitive temperature. In the past, carob was used for sweetening and treating different ailments. Carob is one of the oldest and most beneficial known plants on the earth (Goulas et al., 2016). The cultivation can be done in regions of less rainfall, and have a life of up to 150 years (El Hajaji et al., 2011). Carob has been taken as a cheap source of human and animal nutrition for years due to its different properties (Ramón‐Laca & Mabberley, 2004). Today, carobs are used as pharmaceuticals, cosmetics, and food (Kotrotsios et al., 2012). The whole year fresh green tree of carob has leaves with the thickly single‐layered upper epidermis. These green parts are composed of phenolic compounds in the leaves cells. The carob is a brown pod that is primarily used as a fruit. The carb surface with wrinkles later on ripening gives a leather smooth texture. There are two prime constituents of carbs including pulp and seeds. The pulp is the region in a pod of carob without seeds. However, the pericarp is its upper layer leathery in texture while the inner one is called the mesocarp. Biohydrogen was produced using solid carob waste from the food sector. Several reaction parameters, including pH, catalysis, nitrogen environment, and water supply, are explored for the process, and the yield is compared to glucose as a control substrate (Bahry et al., 2022). Water is used to remove the pods, and microorganisms fermented them. In place of ethylene glycol, a number of ionic liquids are investigated for extraction of dry ethanol. In comparison to nonthermal extraction, microwave‐assisted extraction of phenolics provided better yields (Sanchez‐Segado et al., 2019). Conditions for drying thin‐layer pulp were researched and improved. The quality and yields of drying carob juice is improved using spray drying. To attain the best sensory, antioxidant, and physiochemical qualities, a variety of roasting conditions are investigated. Pods are removed to determine their sugar content, and the extraction is planned using the mathematical Taguchi technique (Huma et al., 2018). In reality, pods are harvested four times in a row. It is discovered that dried ripe pod powder works well as a dye pollution absorber (methylene blue). By reacting with Ce(NO_3_)_3_ 6H_2_O, an aqueous extract of leaves was utilized to create CeO_2_ nanoparticles (NPs) (Tagnamas et al., 2018). These NPs exhibited potent cytotoxic and antioxidant properties. To create ZnO‐NPs, Zn(CH_3_COO)_2_ was treated with an aqueous extract of leaves. Strong cytotoxic action is shown by these NPs against human breast cancer cells (Javadi et al., 2019).

Rats exposed to STZ‐nicotinamide‐induced hyperglycemia were protected by a methanolic extract of dry, unripe pods. Water and ethyl acetate are used to extract the carob honey (El‐Haskoury et al., 2019). Rats with diabetes induced by STZ had a hypoglycemic responses to both extracts. Unripe, dry pods were extracted using 70% aqueous methanol, and the extract was fractionated using dichloromethane and water. Both fractions had an active antidiarrheal and antiemetic in the rat model after being examined for their overall chemical makeup (El‐Haskoury et al., 2019).

Origin, harvesting timing, and cultivars are the main factors on which the chemical composition of carob pulp depends. The total contents of sugar (fructose, glucose, and sucrose) are high in the pulp. Moreover, 185v of cellulose and hemicellulose are also present. The high concentration of condensed tannins (16%–20% of dry weight) is also present in ripened pods of carob (Goulas, 2016).

## 2 NUTRITIONAL AND CHEMICAL COMPOSITION OF CARBS

**FIGURE 1:**
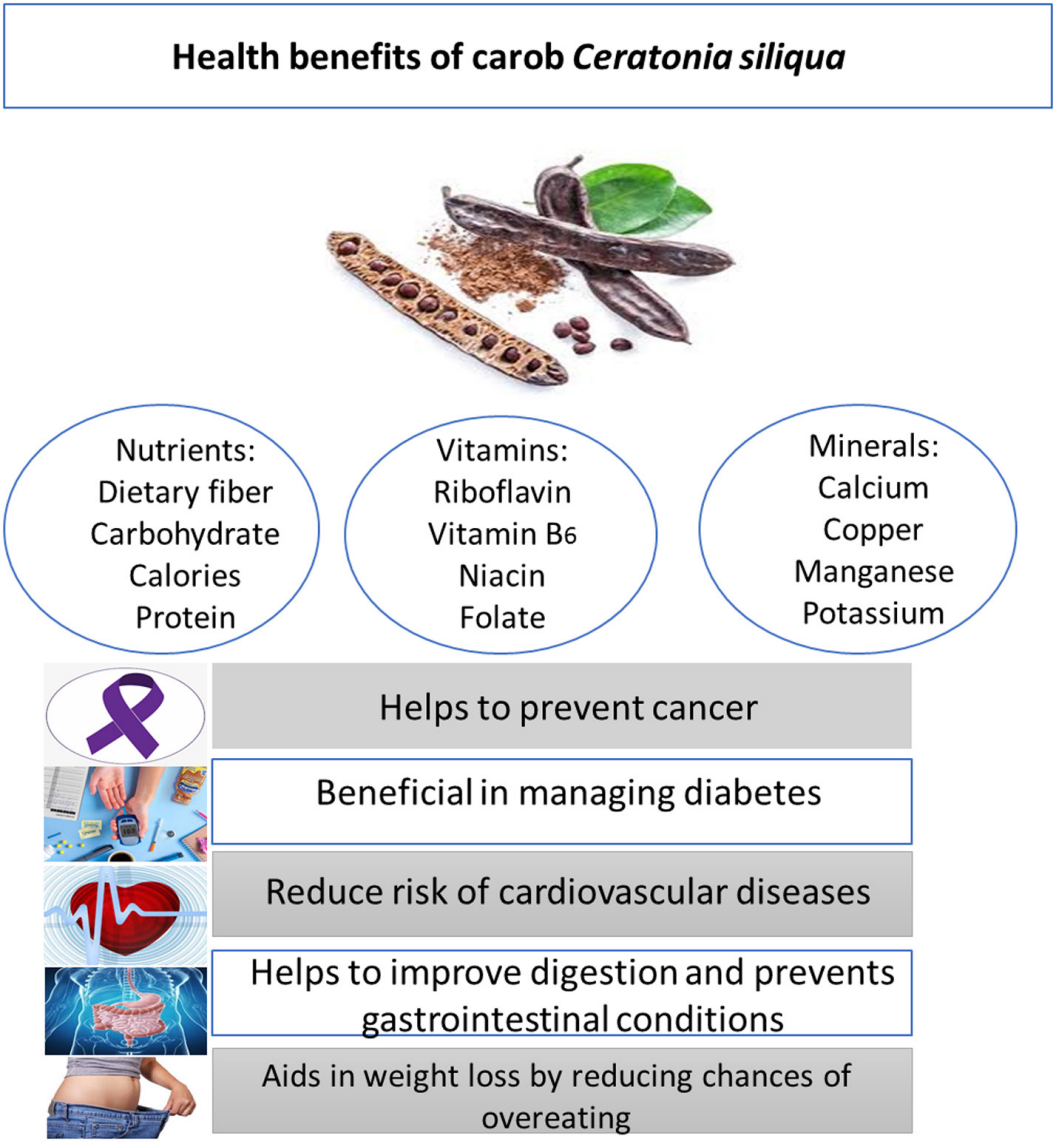
Nutritional composition of carob and health benefits.

The carob fruit is complicated mixture of secondary metabolites. A variety of nutrients including fiber, sugar, and various polyphenols are present. Many minerals and amino acids are also abundant in carob. The nutritional makeup of carob and its health advantages are shown in Figure 1.

### 2.1 Carbohydrates

Carob fruit is a vital source of sugar and has high nutritional value. Previous research revealed that the carbohydrates contents in the cultivated range about 40–55 g 100 g^−1^ dm (Turhan, 2014). Usually, the carob cultivar contains higher sugar content (Sigge et al. 2011). According to sugar composition, the carob, sucrose is vital source of CHO and its quantity is up to 52 g 100 g^−1^ dm (Diaz, 1997). Fructose and glucose concentrations may be as high as 1.8–12.5 g 100 g^−1^ dm and 1.8–10.2 g 100 g^−1^ dm respectively. Carob sugar is taken from the fruit and used to make natural carob syrup. The cutting‐edge method for its recovery to create carob syrup has been established (Livesey, 2003).

### 2.2 Proteins

There are several amino acids present in carob fruits such as sulfur‐containing amino acids (methionine and cysteine), acidic (aspartic acid and glutamic acid), hydroxylic (serine and tyrosine), aliphatic (alanine, glycine, isoleucine, leucine, proline and valine), basic (arginine, histidine, and lysine), and amidic (asparagine and glutamine) (Singh et al., 2007). Around 57% of the amino acid composition in pods is made up of basic and amidic amino groups. Carob fruits were examined as an excellent supply of amino acids in accordance with the World Health Organization's (WHO) requirements for protein. Especially, the concentration of required essential amino acids in carob fruit fairly crosses the WHO standards (Miś & Dziki, 2013).

### 2.3 Minerals

Calcium and potassium are both found in large quantities in carob fruits. Potassium concentrations may reach 970 mg 100 g^−1^ dry weight and 1120 mg 100 g^−1^ dry weight, whereas salt concentrations start at 300 mg 100 g^−1^ dry weight (Rizzo et al., 2004). As 1 L of milk contains 1200 mg of calcium whereas one cup of milk has the same amount of calcium as one piece of the carob fruit. Carob fruit contains little amounts of important bio components like magnesium and phosphorus. Microminerals including manganese, nickel, cobalt, zinc, barium, iron, and cobalt are also included in it. Iron is the micromineral with the greatest concentration. Compared to seeds, pods contain a lower concentration of bio components (Barak & Mudgil, 2014).

### 2.4 Fibers

By removing the water from carob pulp that makes up between 30% and 40% of the total carob pulp. The carob fibers are divided into two categories including soluble and insoluble fibers (Camero & Merino, 2004). The process of obtaining natural fibers of carob have also been licensed (Haber, 2002). Lignin, cellulose, and hemicellulose make up the insoluble portion of dietary fibers. However, the minimal quantity of polyphenols in carob spans the range of 70% of total fibers. The difference between carob fibers and other dietary fiber sources is due to the higher level of polyphenols. Carob fiber is a dietary fiber that is both insoluble and non‐fermentable (Nasar‐Abbas et al., 2016). The soluble fiber is less (maximum 10 g 100 g L^−1^ of that carob fiber) and consists of a simple category of carbohydrates. Ultimately, carob fibers have functions in the rheology of dough in bakery‐based products (Nawrocka et al., 2016).

## 3 PHYTOCHEMICAL COMPOSITION OF CARB

**FIGURE 2:**
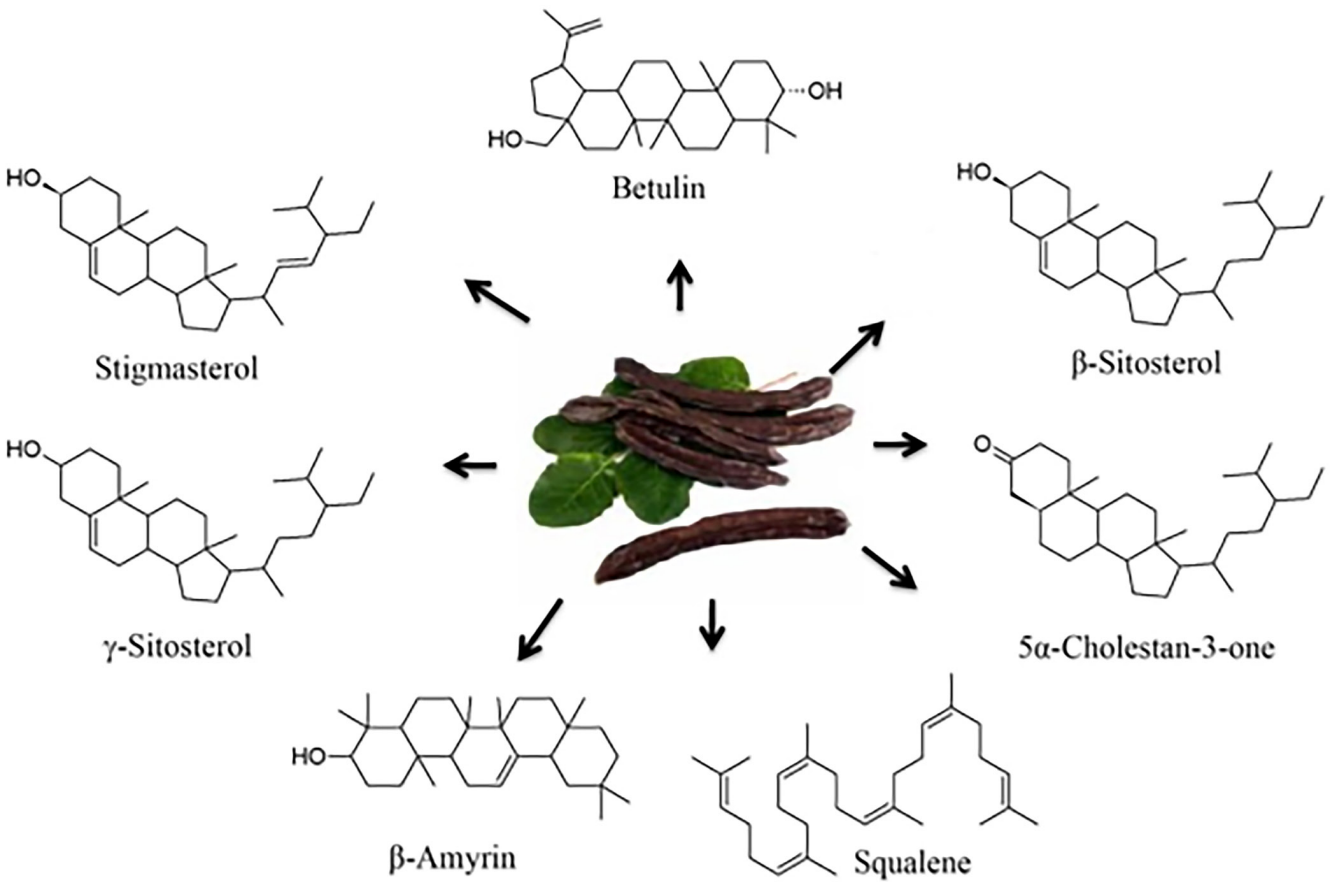
Chemical compositions of carob.

Polypchemical particularly dietary fibers (27%–50%), tannins (18%–20%), carbohydrates and minerals (iron, potassium, sodium, magnesium, zinc, and copper) are present in large quantity while proteins (3%–4%) and lipids (0.4%–0.8%) are present in fewer amounts in carob pods. The fruit is famous due to a high number of sugars which contains important constitutes including fructose (5%–7%), glucose (5%–6%), and sucrose (32%–38%). The large quantity of dietary fibers is also present in pods (Palafox‐Carlos et al., 2011). To identify the presence of polyphenolic compounds, the HPLC method of chromatography was utilized that exposed the existence of non‐hydrolyzed tannins. It is proanthocyanins made of flavan‐3‐ol groups and their galloyl esters, gallic acid, catechin, epicatechin gallate, epigallocatechin gallate, and quercetin glycosides (Ortega et al., 2009). Previous studies suggested that hydrolyzed tannins are also present in carob pods in the form of flavonoids (26%), phenolic acids (cinnamic, *p*‐coumaric, and gallic acids), tannins, hydroxytyrosol, flavone glycosides, and polyphenols (Papagiannopoulos et al., 2004). Electrolytes like sodium, zinc, manganese, potassium, iron, and copper are also found in large quantities in carob juice. The chemical composition of carob pods varies following the climate, specie, maturity stage, and various parts of the tree. Pyrogallol (48.02%–3.55%), catechin (19.10%–2.11%) and plus tannic acid (9.01%–1.40%) are found to be major components in mature pods (Toydemir et al., 2013). Nonetheless, the catechin (16.52%–2.34%), epicatechin (12.26%–1.04%), gallic acid (15.12%–2.31%), pyrogallol (26.45%–3.03%), and chlorogenic acid (15.01%–1.72%) are found in immature pods (Rtibi et al., 2015). Phenols in the form of tannins (13%–0.45%), kaempferol (77%–2.43%), polydatin (0.85%–0.22%), and catechin hydrate (4.30%–0.3%) are present in the leaves are identified. The chemical compositions of carob are shown in Figure 2.

Similarly, it is also revealed that fibers are present richer in leaves than in pods. Carob extracts contain more reduced sugar than leaves (Rtibi et al., 2016).

## 4 BIOAVAILABILITY OF BIOACTIVE COMPOUNDS

**FIGURE 3:**
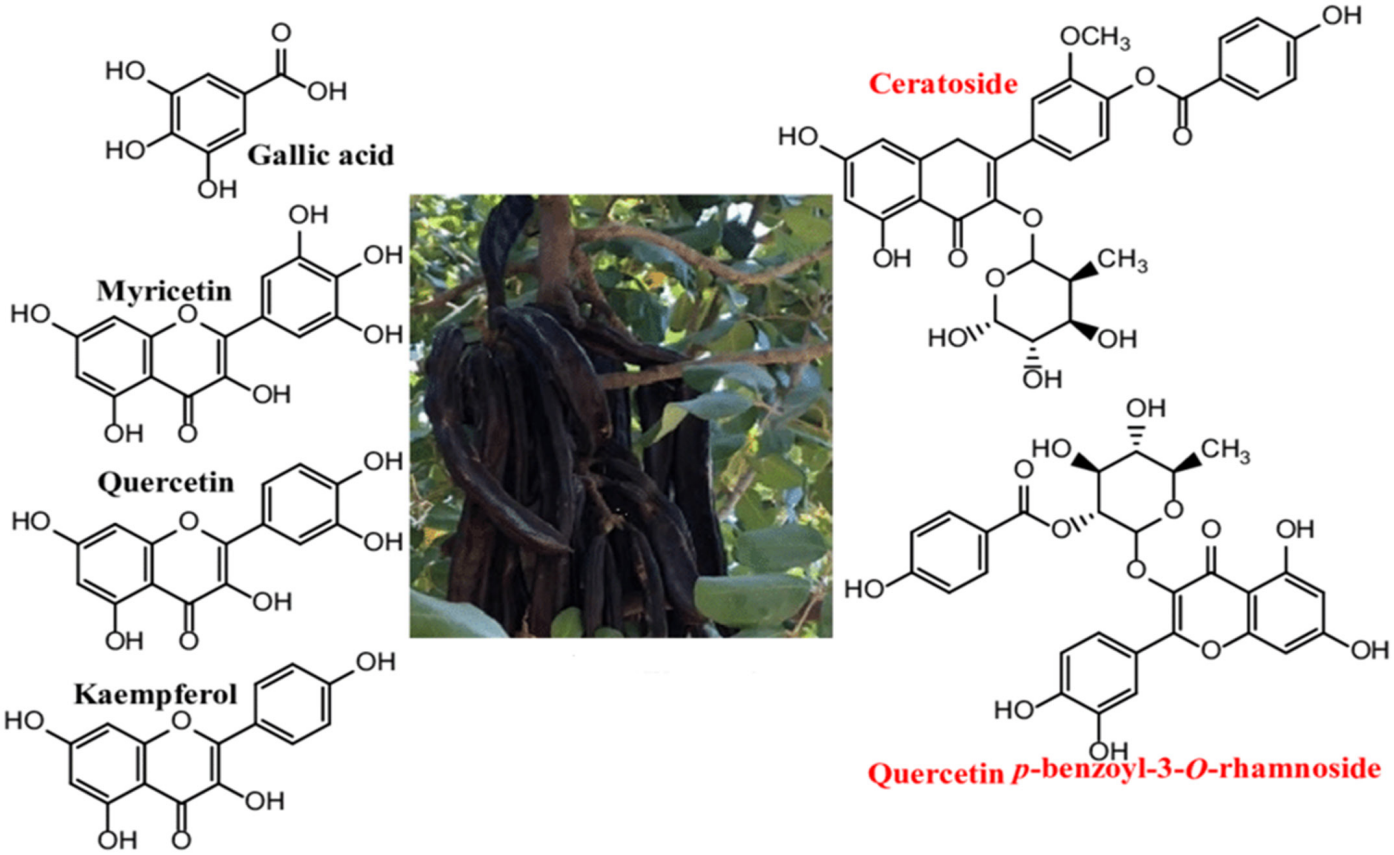
Bioactive compounds of carob.

The biological functions of phenols rely on their deportment in the digestive tract. A few polyphenolic compounds exist in medicinal plants. The sufficient amount of polyphenols is absorbed and transformed to enhance health. Bioactive compounds of carob are shown in Figure 3.

Bio accessibility and their capability of crossing the mucosal barrier of the intestine are the typical factors that define the bioavailability of the constituents (Crozier et al., 2010). The extract of compounds from food matrices and their state of stability in the gastrointestinal condition is known as bioaccessibility (Ali, Manzoor, et al., 2022; Ali, Riaz, et al., 2022; Gawlik‐Dziki, 2012). This procedure of substances relies on different factors encompassing matric composition, initial concentration in the matrix, and the factors related to the host like the presence of enzymes that contributes to the mechanism of digestion and the physiochemical characteristics of the fluids of gastro‐intestine. Moreover, the polyphenols is combined with further ingredients of food like fibers, lipids, and sugars that improve or reduce the bioavailability by process of digestion (Tagliazucchi et al., 2010). The tannins and catechin bioavailability were examined outside the body of rats (small bowel parts). Despite are observed of both components by the intestine. However, catechin is passed through the gut slowly. The less molecular weight phenols like isoflavones and gallic acid superficially get assimilated via the tract (Tagliazucchi et al., 2012). Some bioactive compounds and their health benefits are demonstrated in Table 1.

**TABLE 1:** Health benefits of bioactive compounds present in carob.

Bioactive compounds	Health benefits	Source	Reference
Polyphenols, gallic acid, epigallocatechin, catechin, quercetin, myricetin, kaempferol and rutin	Antioxidant, anti‐inflammatory, antibacterial activities, effective against neurogenerative disorders and antitumoral activities	Carob pulp, carob fiber, carob pod and carob seed extract	Abulyazid et al. (2017); Rico et al. (2019); Rodríguez‐Solana et al. (2019)
D‐pinitol	Anticancer and antidiabetic effect	Carob pulp extract	Christou et al. (2019)
Tannins	Antidiarrheal effect	Carob bean juice	Sigge et al. (2011)
Cinnamic acid	Antioxidant activities and hepatoprotective effect	Carob fruit extract	Dhaouadi et al. (2014); Roseiro et al. (2013)
Galactomannan	Effective in gastrointestinal health	Carob pod endosperm	Xie et al. (2020)
Chlorogenic acid and epicatechin	Reduce intestinal glucose absorption and laxative activities	Carob seed	Abu Hafsa et al. (2017)
Flavonol glycosides	Increased lipid metabolism and reduce LDL level	Carob pulp extract	Gruendel et al. (2007)

### 4.1 Carob polyphenols and their bioavailability

The carob fruits are high in phenolic acid that is compressed by gallotannins and flavonoids (Benchikh et al., 2014). Although polyphenols represent a broad range in in vitro biological properties, and their bioavailability reduces in vivo efficiency. However, they are the most abundant in the human diet and their behavior is not always parallel to the abundance due to poor absorption or rapid removal (Chahar et al., 2011). Polyphenols have a bioactivity of 10% (animal tests) with a range of around 2%–20%. It is extremely necessary to have a way of increasing their bioavailability because a broad sample of participants is needed in clinical trials to illustrate the efficacy and thus such tests are not affordable (Serafini et al., 2010). The first strategy should be to investigate the bioavailability as well as pharmacokinetic profile because these are the major factors causing its action. A lot of therapeutic potential phytochemicals have a high in vitro record but decreased in vivo due to their weak ADME (absorption, distribution, excretion, and metabolism) properties. The fundamental parameters to define a pharmacokinetic profile to an appropriate degree are *T*_max_, *C*_max_, *t*_1/2_, and AUC. *C*_max_ is the maximum reached the amount of medication after one dose and after the second dose; *T*_max_ is the time taken to achieve the maximum concentration of a medication (*C*_max_); *t*_1/2_ is the time for the medicine to achieve half the initial concentration; whereas AUC (the region below the curve) is referred to as the cumulative concentration of a drug over time (Lu et al., 2014). A more detailed evaluation of bioavailability in human and animal studies is needed for the low absorption as well as intensive metabolism of compounds with poor bioavailability (Cao et al., 2015). The number of research to quantify in vivo flavonoids was focused on LC–MS/MS platforms. Assessing their persistence in human plasma using novel pharmacokinetic techniques will provide the foundation on which scientists can build to resolve the impediment of poor bioavailability. In vivo studies, further work should be carried out on molecules with impressive outcomes as the distance between two situations is often impossible to overcome (Wong et al., 2014).

## 5 FOOD APPLICATIONS OF CAROB

**FIGURE 4:**
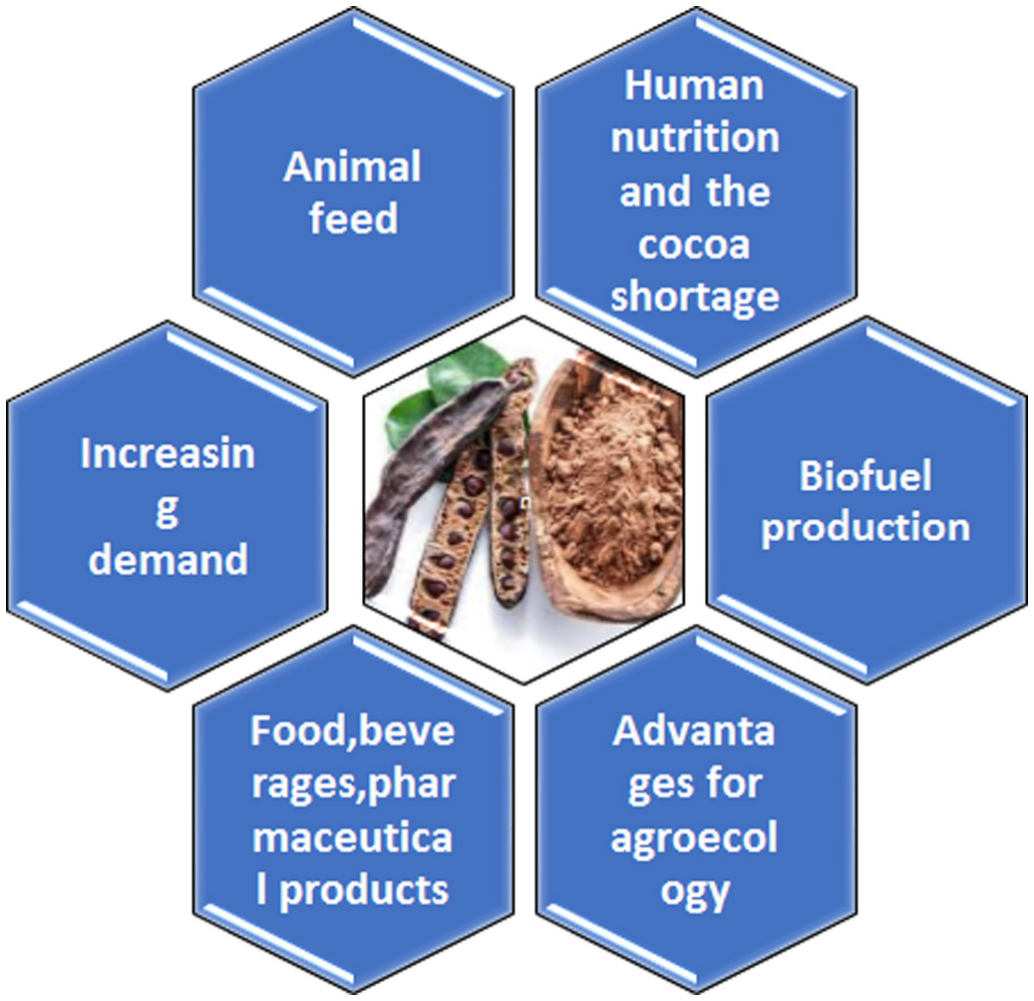
The use of carob.

Nowadays, carob seeds are being used as an alternative to cocoa powder in food items and scrutiny on comprehensive examination of chemical and sensory attributes of products (Srour et al., 2016). Currently, the replacement of cocoa in white milk chocolate with carob pod powder has been investigated. Protein contents were increased to a little extent by utilizing more carob powder instead of cocoa in white chocolate, but the fiber and sugar content upgrades highly. Some uses of carob are shown in Figure 4.

By increasing the concentration of carob powder in white chocolate, the calcium, potassium, magnesium, and sodium concentrations were investigated to high whereas iron and zinc concentrations was found to drop (Salem & Fahad 2012). The most important impact was the stepwise reduction of caffeine, which completely vanished at 100% concentration of powder carob. The aroma did not show any special difference by increasing carob flour quantity (Tsatsaragkou et al., 2012). The results demonstrated that nutritional status, sensory and functional properties are improved by using carob in milk chocolate. It has been understood as the best alternative to cocoa powder in the synthesis of products based on chocolate. In the manufacture of cakes that are free from gluten to banana and soy flour. The impact of carob flour was also studied by investigating the sensory and physicochemical characteristics of cakes of different formulations (Haber, 2003).

The calories, lipids, and carbohydrates are lowered while the concentration of dietary fibers increases by using carob powder in cakes. But the resilience, elasticity, and cohesiveness in cakes are lessened by the use of carob flour instead of cocoa powder. The cake with 75% replaced with carob powder that have no noticeable differences in sensory properties compared to cocoa‐based cakes (Rosa et al., 2015). The manufacture of cakes that are free of gluten have fewer calories and high in fiber and protein content. Its pleasing sensory attributes for celiac‐suffering patients. A substitute for chocolate milk carob relying on milk beverages by using carob powder is also manufactured (Iipumbu, 2008). The manufacture of different carob‐based beverages and powders is done by utilizing both unroasted and roasted pulp of carob of various varieties.

The roasted powder shows richness of antioxidant activity and phenolic contents and results. Moreover, the roasted powder enhanced the acceptability aspects of sensory properties carob coffee odor, flavor, caramel aroma, mocha odor, mouthfeel, viscosity, and afterward bitter taste (Moreira et al., 2017). It may be due to the association of pyrazines, pyridine, ketones, and aldehydes that are concerned with typical roasted taste and aroma (Afoakwa, 2016). Some functional food items are mentioned in Table 2.

**TABLE 2:** Carob‐enriched functional foods and their impact on quality of product.

Carob‐based food products	Positive impact	Reference
Bread fortified with carob pod flour	Bread quality improved, high antioxidant and phenolic content, and improve organoleptic attributes	Hoehnel et al. (2019)
Carob spread	Improved texture and color attributes, and good source of minerals	Aydın and Özdemir (2017)
Carob powder‐based milk beverages	Highest phenolic content, antioxidant activities, and improved color, taste texture, and overall acceptability	Srour et al. (2016)
Rice based snacks enriched with pea and carob fruit powder	Improved organoleptic and textural attributes, and high phenolic and antioxidants compounds	Arribas et al. (2019)
Carob powder‐based yogurt	Produce low‐fat yogurt, high fiber content, produce low lactose, and increased sweetness	Moreira et al. (2017)
Carob powder‐based gluten free cakes	Improved dietary fiber content, lower caloric content, rich in protein, decreased cohesiveness, and good sensory attributes	Rosa et al. (2015)
Carob flour‐based pasta	High phenolic contents, higher antioxidant capacities, higher glycemic index (GI), and improved sensory attributes	Arribas et al. (2020)
Carob syrup used as sugar replacer	Increased fermentation, reduce microbial activity, and higher yield of mannitol production	Nasar‐Abbas et al. (2016)
Edible coating of carob bean gum‐based products	Improve physical appearance, increased shelf life, minimize the losses of bioactive compounds, and reduce oxidation process	Rojas‐Argudo et al. (2009)
Carob bean gum‐based bakery products	Increase rheological properties, improve yield of bakery items, and increased water absorption capacity	Zhu et al. (2019)
Carob‐based ice cream	Used as stabilizer, decrease melting point, and increase viscosity	Bahramparvar and Tehrani (2011)

## 6 BIOCHEMICAL APPLICATIONS OF CAROB

### 6.1 Antimicrobial and antifungal activities

*Ceratonia siliqua* methanol extract was tested for antimicrobial activities as contrasted to Plantago major methanol extract which was more effective for many of these bacteria. *C. siliqua* extract was more active in Enterococcus sp. Methanol and aqueous extracts were tested alone and in conjunction with many other antimicrobial agents (gentamicin, amikacin, ampicillin, and clindamycin) (Talibi et al., 2012). Extracts and antimicrobial agents combined were more successful than each independently. Antibacterial property against *Pectobacterium atrosepticum* was evaluated in potato soft red. The acetone extract was more active. It was found that the methanol extract of the leaves was active for *Listeria monocytogenes*. HPLC extract analysis provided seven antibacterial compounds and extract dry pods were screened for 14 forms of fungi and bacteria (Meziani et al., 2015).

Dried plant powder was absorbed into methanol, and their mixture was centrifuged, filtered, and screened for antifungal and antibacterial activity. The photosensitivity has also been investigated and proven to be adequate. It was found to be highly efficient against 11 of them in 1000 and 500 mg mL^−1^ amounts. Chloroform as well as hydroalcoholic (no indication of ratio) extracts of dry particles were processed and found to be active toward 15 fungal and bacterial organisms including three species of *Caucasian albania* (Aissani et al., 2012).

Hexane, ethyl‐acetate, chloroform, and methanol extracts from dry leaves were made and evaluated against citrus‐sour‐rot agent *Geotrichum candidum* for antifungal activity (Rahmoun et al., 2014). The hexane and chloroform were more inactive, whereas the methanol extract was more active than the ethyl acetate extract. It was militant against everybody. The total phenolic level was calculated to be 465.5 mg g^−1^ whereas gallic acid and the total flavonoid was found to be 24.6 mg g^−1^. The extract has been classified as antimicrobial properties and moderate antioxidant (DPPH). N‐hexane, ethyl acetate, and water have been extracted from dry leaves (Al‐fawwaz & Al‐Khaza'leh 2016).

### 6.2 Antidiabetic activities

The dry pod extract of ethanol/water has been studied for streptozotocin‐induced diabetes in rats. The blood glucose was found to be decrease (Hsouna et al., 2012). The combination of dried Roselle flowers (*Hibiscus sabdariffa*) and dry carob pods was extracted from water and administered to diabetic alloxane‐induced rats. The extract has been checked both with and without gamma rays combination of plant powder (Jamous et al., 2015).

In alloxane‐induced diabetic rats, an aqueous extract of the premature pod was evaluated for antidiabetic. This is more successful than an aqueous extract from mature pods. Powder from dry pods for phytosterols with n‐hexane was isolated (Mokhtari et al., 2011). The same party used the same extract for the study of the same disease except female rabbits are not pregnant. Fiber‐diluted aqueous extract of seed‐free dry pods and was prepared and tested to suppress the disease of the antidiabetic by inhibiting glucosidase. The appropriate activity has been found (Hamza & Al‐Seeni, 2015).

### 6.3 Antioxidant activities

Ethanolic leaves and pulp extracts (all sexes of the tree) have been screened for radical (DPPH) as well as antioxidant activities and extract leaves were observed to be more active. Dichloromethane, hexane, diethyl ether, methanol/water (8:2 v/v), and ethyl‐acetate have been extracted successively from the leaves. Extracts from the three tree varieties have been examined for antioxidant activity (DPPH) and total phenolic content (Rtibi et al., 2017).

Antioxidants, carotenoids, and polyphenols were evaluated from ethanol extract of dry pods. Eighty percent aqueous methanol extract was ready and measured on antioxidant activity (three methods). However, HPLC reverse stage analysis was performed for this purpose (Mounce & Al‐Saeed 2017). The pods (no seeds) were extracted using water, petroleum ether, methanol, ethanol, hexane, and acetone. Extracts were tested for total phenolic content, antioxidant activity (ABTS), and cerebral and myocardial lipid peroxidation in vitro and in vivo (rats). Polar extracts have been more active than non‐polar extracts.

Methanol extract from leaves was tested by various methods for antioxidant activity and was found to be very effective as compared to many other fruit plants. The total phenolic content and the antioxidant (DPPH and FRAP) ability of methane extract from dry leaves were identified. Compared to other plants studied, there were moderate activities in carob. Aquatic extract of dry carob pods was prepared and its total phenolic content and antioxidant activity were established. Both aqueous and methanol extracts have been found fairly high (plant component not indicated) and have been checked for antioxidant activity as well as total phenolic content (Macho‐Gonzalez et al., 2017).

### 6.4 Anti‐cancer effects

Colorectal cancer has become one of Western society's most common cancers (Johns & Houlston, 2001). Epidemiological and clinical studies have shown that a regulatory diet can suppress colorectal cancer. Researchers conclude that various phenolic compounds are highly promising associated substances (Nayak et al., 2015) for fruits, vegetables, rice, tea, and wine, which are very useful for the body of humans. The purpose is to minimize oxidative stress by chelating free radicals or redox activity. Some studies have also shown that the proliferation of different cancer cell line forms can be effectively inhibited (O'Keefe, 2016). Dietary fiber is another possible factor that can reduce the risk of colon cancer. High‐fat and high‐protein foods have a beneficial impact on colorectal cancer but have a detrimental relationship to high‐complex carbohydrates and high in fiber intake (Ali, Mughal, et al., 2021; Klenow et al., 2008). Earlier studies found that polyphenols and dietary fiber would reduce the risk of cancer, while carob fiber combines with those two nutrients (Klenow et al., 2009).

### 6.5 Anti‐reflux effects

Reflux in infants is normal. In 77% of infants under the age of 3 months, regurgitation was reported at least once a day. Carob bean gum is the most widely used milk thickener in European countries. Studies have shown that the number of carob bean gum regurgitation episodes decreases significantly and enhances other symptoms, such as crying, sleep disturbance, and gastroesophageal reflux (O'Keefe, 2016). Researchers investigated the effect on reflux as well as tolerance indicators in an adolescent gastroesophageal expression of carob bean gum thickened formulas. In the study, 56 qualified infants (1–6 months of age) were randomly assigned to either 0.33 g 100 mL^−1^ (Formula A) or 0.45 g 100 mL^−1^ (Formula B) of cold‐lösable gum bean or 0.45 g 100 mL^−1^ (Formula C) of hot‐lösble gum for 2 weeks. Results have shown that formula A (i.e., 0.33 g 100 mL^−1^) effectively decreased some pH‐monitoring levels of unpretentious gastroesophageal reflux, higher body weight, and well‐tolerated childhood. Thirty‐nine babies with three or more regurgitation episodes a day were studied in another study (Nayak et al., 2015). Besides, gastric emptying efficiency of different milk formulations was measured. The formulas are A, B, and C with 0.35 g of carbon beans per 100 mL^−1^ (HL‐350) and 0.45 g per 100 mL^−1^ (HL‐450) as well as (HL‐00), respectively. The results indicated that the HL‐350 or HL‐450 treatment of infants is greater than that of HL‐00 (reflux episodes). A comparison of two formulations revealed that HL‐450 had a slower growth rate of gastroesophageal reflux gastric emptying in children. In a further study, the authors proceeded to analyze the effect of the formula containing the bean gum (HL‐350 in younger infants), which can be used as a thickening formula of infant formula, significantly reducing the number of reflux episodes in babies and young children (Klenow et al., 2009).

### 6.6 Anti‐diabetic effects

The anti‐diabetic effects of carob‐containing herbal preparations and other natural products have been tested. The formulations were used as herbal remedies for people with diabetes who had a low glycemic index (Milek et al., 2015). The Carob gum diet has shown a drop in rat blood glucose levels. Dos Santos et al. further estimated in vitro the glycemic index of carob flour at 40.6. D‐pinitol may be present in carob products for anti‐diabetic properties, as it controls blood sugar levels in Type II diabetes mellitus patients by enhancing insulin sensitivity (Son et al., 2010). Carob syrup is viewed as a major source of D‐pinitol. Ten grams of it is adequate to decrease the sugar level in type II diabetes as compared to the normal dose (10 mg D‐pinitol/kg body weight). Tetik and Yüksel (2014) proposed that D‐pinitol could have strong measures with insulin, and therefore improve glycemic control by evaluating its effectiveness in diabetes animal models. The study also reported that D‐pinitol induced increased glucose absorption in the L6 line of muscles, indicating its presence in the glucose metabolic pathway of muscles rather than the increased development or action of insulin (Ali, Ain, et al., 2021; Coviello et al., 2015).

### 6.7 Anti‐diarrheal effects

The different percentages of carob are reduced the signs of diarrhea. The previous study has shown that 2% carob solution can prevent hemagglutination and adherence of *Escherichia coli* on specific epithelial cells of the intestines. The efficacy of the proposed fraction may be demonstrated by blocking the adherence of bacteria isolated from the highest small intestinal tract of children. The proportion consisted of 40% tannins or 21.2% polyphenols, and 26.4% dietary fiber. Tannins are not held responsible for anti‐diarrheal intervention for the first time. Liu et al. (2014) demonstrated by in vitro and in vivo models strategies that the tannin extraction of rhubarb downregulates the pathways PKA/p‐CREB and consequently influences water transport within the cells of aquaporins 2 and 3. Wursch (1991) also patented an anti‐diarrheal dietetic drug, which includes water‐insoluble tannins of at least 20% by weight by weight of particulate carob pods based on dry matter.

### 6.8 Anti‐hyperlipidemia

Effects of high blood lipid or lipoprotein levels can cause atherosclerosis and later vascular and heart disease. The supplements that reduce the amount of lipids and cholesterol in the blood. Patents have been given for a cholesterol‐reducing preparation consisting of at least one dietary fiber picked from the carob‐fruit flesh group. In addition, the key ingredients for proprietary foodstuffs are carob fiber in combination with n‐3 fatty acids that benefit cardiovascular safety. The effects of carob constituents in hyperlipidemia have been extensively studied in vivo and in vitro models. LBG has been demonstrated to reduce cholesterol as well as lipid levels in rats. However, LBG had no significant impact on the cholesterol as well as triglycerol levels of diabetic rats in a more recent study (Yamamoto et al., 2000). The carob fruit findings are less controversial. Carob powder was applied to Sprague–Dawley rats together with a hyperlipidemic intake to dose‐dependent cholesterol as well as triglycerides. The findings also indicate that in carob powder ingested, the histopathological status of the heart, as well as the kidney of the animal while rats who adopted the hyperlipidemic diet had extreme disorders. Carob powder may be a possible choice for an overweight or obese diet (Hassanein et al., 2015). Valero‐Munoz et al. found that carob pod fibers have blocked physiological events contributing to atherosclerosis in rabbits with dyslipidemia. The function of SIRT1 and PGC‐1a, proteins that play a key role in the vascular as well as metabolic processes (Valero‐Munoz et al., 2014).

## 7 CAROB AND CLINICAL TRIALS

The clinical trials on carob is represented the use carob‐containing mixtures along with many other substances (Vandenplas et al., 2013). Results showed that only carbohydrates often include child hypercholesterolemia, regurgitation, and diarrhea. The majority of studies were performed on other diseases like cancer and diabetes. The outcomes of treatment for conditions such as hypercholesterolemia as well as diarrhea can be quickly discerned and control can be demonstrated over a shorter time period.

### 7.1 Infant regurgitation

The effectiveness of two ARFs for the therapy of infant regurgitation was evaluated and compared (Vandenplas et al., 2013). The ARF‐1 and ARF‐2 included LBG with a double‐blind cross‐over test in two groups of infants carried out for a month. For all classes and specific anthropometric criteria, the net number of babies was 115 with an average age of 9.1 weeks. ARF‐2 was systematically more successful than ARF‐1 and the total number of regurgitation episodes decreased from 8.25 to 2.32 in ARF‐1 as well as to 1.89 in ARF‐2. The same change was found for the mean volume of regurgitation with stronger action from ARF‐2.

Both ARF‐1 and ARF‐2 showed effectiveness in reducing infant volumes and the percentage of infant regurgitations that providing a broader spectrum of treatment options. Maiti et al. (2010) studied the results in infants with episodes of regurgitation of two formulae involving LBG at various concentrations. Thirty‐nine children with far more than three regurgitation episodes a day were investigated in this study. A comparison of the two milk‐based solutions in gastric emptying was measured at some times during feeding. The first solution contained LBG 0.35 g 100 mL portion 1 (HL‐350), whereas the second one contained 0.45 g 100 mL portion 1 (HL‐450). The best combination (HL‐00) was, on the other hand, clean of LBG. The impact assessment on regurgitation events was performed in 27 babies with episodes randomly allocated for 1 week with HL‐00 to obtain the formula. Results show that regurgitation seasons for infants who were fed HL‐350 or HL‐450, instead of HL‐00, were substantially lower.

Comparisons of the two formulas demonstrated that in infants with gastric esophageal reflux, HL‐450 showed a lower capacity of gastric emptying. Miyazawa et al. (2004) have examined the impact of both LBG‐based formulations at various rates in infants with episodes of regurgitation in earlier studies. Formula HL‐350 has shown promising promise by reducing episodes of regurgitation in 4‐month‐old children with reflux. For this study, researchers investigated the impact of HL‐350 for infants below 2 months. For this reason, a 2‐week study allocated 20 children with more than three regurgitation episodes a day. The babies were divided into two groups and the first was fed HL‐350 in the first week, and the second was controlled milk (HL‐00). The second party followed the opposite order. The number of regurgitation episodes in infants was substantially decreased during the week when they fed HL‐350 compared to the week of milk control. In addition, HL‐350 did not affect the delay in gastric acid secretion. The care of gastrointestinal bleeding in babies was performed successfully using LBG (Panghal et al., 2014).

## 8 ECONOMIC IMPACT

**FIGURE 5:**
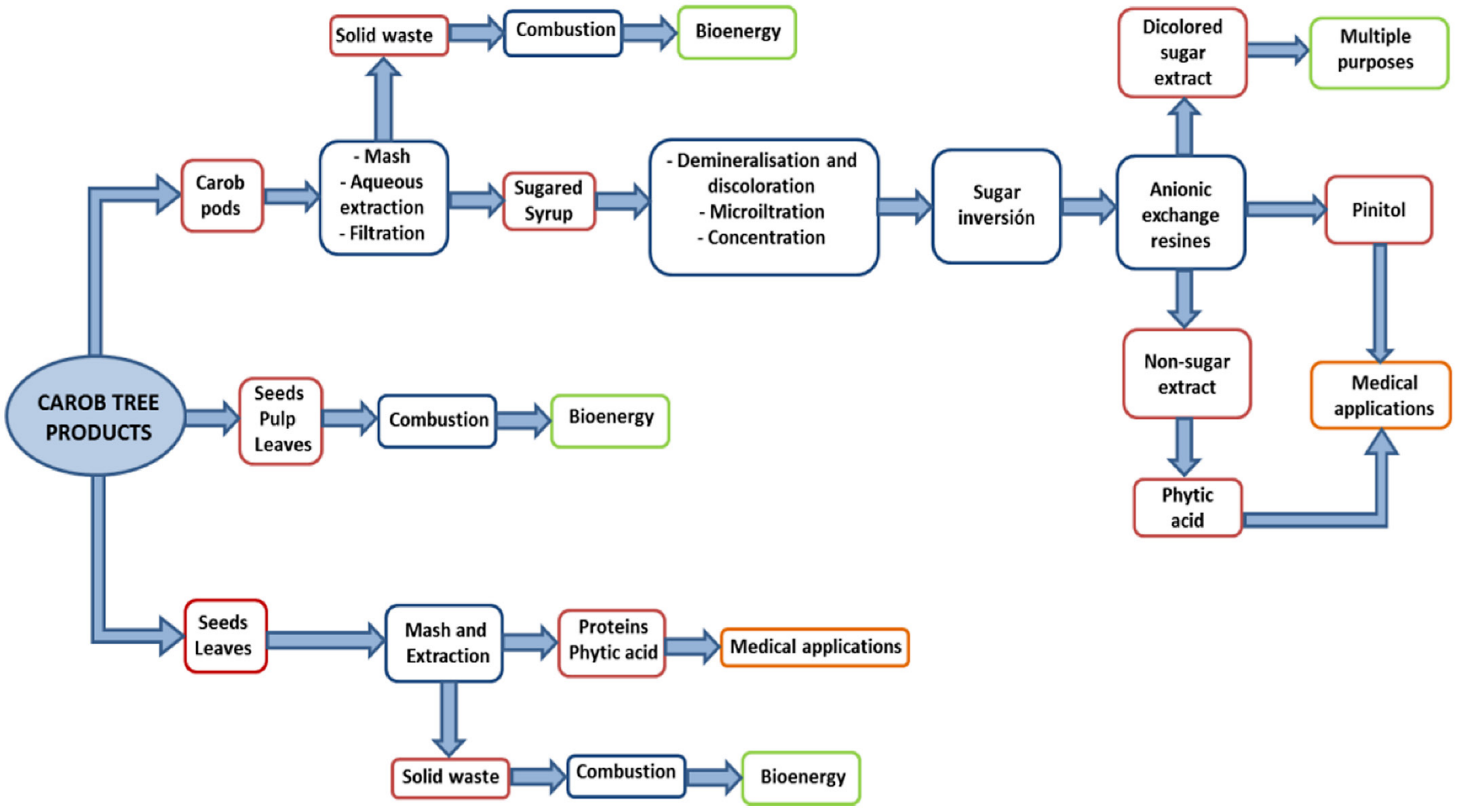
Carob tree products.

The harvesting of the carob tree seems to be great economic and environmental significance for the Island of Cyprus. The carob tree is regarded as the main crop for the long‐term survival of its high natural farms in terms of the climate. On the other hand, the high production of carob is well contributes to the local economy. In the last few decades carob has been recognized as the “black gold of Cyprus” due to financial value. However, it is the largest agricultural export. Carob production is currently substantially rising in Cyprus, because it is no longer viewed profitably. Carob fruit is mostly used to produce endosperm to the food industry in particular for the production of carob syrup. Similarly, the carob seed leaves are used for bioenergy and as animal feed as shown in Figure 5.

The carob crops is able to deliver viability for production taking into account the health impacts of carob. Carob fibers and polyphenols are not used but pharmacological studies have related them to important health‐promoting properties. These compounds also become components in modern functional foods, dietary supplements, and plant‐based medications. Research is also required for the utilization of LBG as a drug carrier and D‐pinitol as an active compound for the treatment of diabetes. Ultimately, the use of such drug applications is utilized to achieve high‐value‐added goods from carob fruit (Goulas et al., 2016).

## 9 CONCLUSION

It is concluded that carob can be included in the regular diet of humans because it is nutrients dense plant source. In addition, it contains enough vegetative fat, protein amino acids, and minerals. However, carob can be used as a natural ingredient for producing fresh, nutritious foods that is depended on the manufacturing process. Carob is basically native to Cyprus that has a high biological value and needs to be protected. As a natural product, carob is not only beneficial to human health but also economically and environmentally important. The results of clinical trials showed that carbs can treat different diseases including hyperlipidemia, diabetes, colorectal cancer, and irritable bowel syndrome.

## AUTHOR CONTRIBUTIONS

**Ali ikram:** Conceptualization (equal). **khair‐ul‐Wajeeha Zafar:** Data curation (equal). **Muhammad Faizan Afzal:** Software (equal). **Afifa Aziz:** Validation (equal). **Izza Faiz ul Rasool:** Visualization (equal). **Ammar AL‐Farga:** Investigation (equal).

## FUNDING INFORMATION

The authors did not receive support from any organization for the submitted work.

## CONFLICT OF INTEREST STATEMENT

The authors declare no conflict of interest.

## ETHICS STATEMENT

There is no need for ethical approval as this was a review article.

## Data Availability

Data used during the current study are available from the corresponding author.
